# Improving the Performance of Outbreak Detection Algorithms by Classifying the Levels of Disease Incidence

**DOI:** 10.1371/journal.pone.0071803

**Published:** 2013-08-19

**Authors:** Honglong Zhang, Shengjie Lai, Liping Wang, Dan Zhao, Dinglun Zhou, Yajia Lan, David L. Buckeridge, Zhongjie Li, Weizhong Yang

**Affiliations:** 1 Key Laboratory of Surveillance and Early-warning on Infectious Disease, Division of Infectious Diseases, Chinese Center for Disease Control and Prevention, Beijing, China; 2 Institute of Immunization and Prevention, Beijing Center for Disease Control and Prevention, Beijing, China; 3 West China School of Public Health, Sichuan University, Chengdu, China; 4 Surveillance Lab, McGill Clinical and Health Informatics, Department of Epidemiology and Biostatistics, McGill University, Montreal, Quebec, Canada; 5 Chinese Center for Disease Control and Prevention, Beijing, China; Tulane School of Public Health and Tropical Medicine, United States of America

## Abstract

We evaluated a novel strategy to improve the performance of outbreak detection algorithms, namely setting the alerting threshold separately in each region according to the disease incidence in that region. By using data on hand, foot and mouth disease in Shandong province, China, we evaluated the impact of disease incidence on the performance of outbreak detection algorithms (EARS-C1, C2 and C3). Compared to applying the same algorithm and threshold to the whole region, setting the optimal threshold in each region according to the level of disease incidence (i.e., high, middle, and low) enhanced sensitivity (C1: from 94.4% to 99.1%, C2: from 93.5% to 95.4%, C3: from 91.7% to 95.4%) and reduced the number of alert signals (the percentage of reduction is C1∶4.3%, C2∶11.9%, C3∶10.3%). Our findings illustrate a general method for improving the accuracy of detection algorithms that is potentially applicable broadly to other diseases and regions.

## Introduction

Detecting infectious diseases outbreaks at an early stage is crucial for timely implementation of control measures, which can minimize morbidity and mortality. A considerable amount of research has focused on developing statistical methods to identify aberrations in disease incidence data accurately and quickly [1]–[4]. In current public health practice, aberrancy-detection algorithms, including temporal (e.g., Exponentially Weighted Moving Average and cumulative sum), spatial (e.g., Spatial Scan Statistic), and spatio-temporal (e.g., Space-Time Scan Statistic) methods, can contribute important information to support outbreak detection and management [3]–[6]. Evaluations of surveillance systems have demonstrated that many factors affect the accuracy of aberration detection, including the mode of disease transmission, seasonal patterns in disease incidence, the detection algorithm used, and the alerting threshold of the algorithm [7], [8]. Disease incidence can vary greatly between regions under surveillance, but it is not known to what extent this variation in incidence affects the accuracy and timeliness of aberration detection if the same algorithm with a constant alerting threshold is applied to all regions.

Hand, foot and mouth disease (HFMD) is caused by serotypes of enterovirus and, usually leads to mild symptoms, but can result in serious complication or death [9]–[11]. In China, HFMD tends to infect infants and children younger than 5 years old [12], with more than one million cases reported and three hundred deaths nationwide in 2009 [13]. Within China, Shandong province is one of the most seriously affected regions with an annual incidence of 92.2 per 100,000 to 149.4 per 100,000 between 2009 and 2012. Early detection of outbreaks and prompt diagnosis and treatment of cases at high risk of severe disease are key principles in minimizing the impact of HFMD [14]. In this study, we used data from surveillance of HFMD in Shandong province to evaluate a novel strategy to improve the accuracy and timeliness of outbreak detection. More precisely, we examined whether setting the optimal alerting threshold separately in each region according to the disease incidence in that region would improve the accuracy and timeliness of outbreak detection as compared to using a constant alerting threshold across all regions.

## Methods

In China, a probable HFMD case was defined as a patient with papular or vesicular rash on hands, feet, mouth or buttocks, with or without fever. A confirmed case was defined as a probable case with laboratory evidence of enterovirus infection (by EV71, CA16, or other enteroviruses) detected by reverse-transcriptase polymerase chain reaction (RT-PCR), real-time RT-PCR, or by virus isolation [15]. HFMD has been a notifiable infectious disease in China since May 2008. Clinicians are required to report both probable and confirmed HFMD cases through a web-based reporting system, the Nationwide Notifiable Infectious Diseases Reporting Information System. Using this system, clinicians in all health care institutes throughout the country can report cases of notifiable infectious diseases via the Internet to a data center located in the Chinese Center of Disease Control and Prevention (China CDC). Cases of HFMD reported between January and December 2009 in Shandong province of China were used in this study.

We obtained the population of the 142 counties in Shandong province in 2009 from Chinese State Statistics Bureau. The counties were categorized into 3 groups on the basis of annual HFMD incidence, ① low level: with the disease incidence rate ranging from 7 to 149 per 100,000, ② middle level: with the disease incidence rate ranging from 150 to 249 per 100,000, ③ high level: with the disease incidence rate ranging from 250 to 420 per 100,000. Following an examination of the distribution of disease incidence by county, we selected these categories to reflect natural groupings in the data.

We used the three Early Aberration Reporting System (EARS) algorithms (C1, C2, and C3) developed by the US Center for Disease Control and Prevention [3], [16]. These algorithms require few historical baseline data and are based on statistical process control methods. These algorithms estimate the expected value on any given day as the average of the observed values over 7 previous days. For the C1 algorithm, the baseline is the past 7 days (ie, t-1 to t-7), while for the C2 and C3 algorithms, the baseline incorporates a 2-day lag before the current day (ie, t-3 to t-9). The C3 algorithm also maintains a 3-day running sum, and the commonly used threshold for C1 and C2 is 2.0 [3], [16]. All three algorithms are described in detail elsewhere [3], [16], [17]. In our study, acknowledging that the algorithm threshold could impact outbreak detection performance, we tested 30 candidate threshold values (from 0.1 to 3.0, interval is 0.1) for C1, C2, and C3, so as to determine the optimal threshold for each algorithm when were applied to data for each incidence category. All algorithms and analyses were implemented with R software [18].

In China, the definition of reported HFMD outbreak was that ≥10 cases occurring in the same gathering settings (e.g., kindergarten, school), or ≥5 cases occurring in the same village or community within one week [19]. The HFMD outbreaks reported in 2009 were assumed to be the only true outbreaks in the data, as all of these outbreaks were verified through field investigation by local public health departments [15]. We defined the start and end of an outbreak as the first and last dates, respectively, of reported cases associated with the outbreak.

We evaluated algorithms in terms of their sensitivity, specificity, and time to detection (TTD). Sensitivity was defined as the number of outbreaks during which at least one alert was signaled, divided by the total number of reported outbreaks. Specificity was defined as the number of non-outbreak days with no alert, divided by the total number of non-outbreak days [20]. TTD was defined as the median number of days from the beginning of each outbreak to the first alert during the outbreak. If the algorithm alerted on the first day of an outbreak, detection time was zero. To enable the calculation of detection timeliness of all outbreaks, if an outbreak was undetected, TTD was assigned the total duration of the outbreak, so as to enable calculation of the median timeliness across all outbreaks. Therefore, TTD is an integrated index that reflects both the timeliness and sensitivity of an algorithm [7]. The optimal threshold for an algorithm was the one with the shortest TTD, or with the highest specificity when (a) the TTD was either same or (b) had a difference of less than half a day and the difference between the specificity was >5.0% [7]. We used the Student t test to examine whether the number of signals was significantly different by setting the optimal threshold in each region according to the level of disease incidence and using the same optimal thresholds to the whole region.

## Results

In 2009, a total of 138,593 cases and 108 outbreaks of HFMD were reported from the 142 counties of Shandong province. The county incidence rate ranged from 7 per 100,000 to 420 per 100,000. According to our classification criteria of low, middle and high disease incidence, there were 85 (59.9%) counties with a low disease incidence, 39 (27.5%) counties with a middle disease incidence, and 18 (12.6%) counties with a high disease incidence (Table 1). The total number of outbreaks reported was 32, 47 and 29 in low, middle and high disease incidence regions, respectively. The median number of cases per outbreak was similar among the three groups with low, middle and high level of disease incidence.

**Table 1:** The cases and outbreaks in the regions with high, middle and low level of hand, foot and mouth (HFMD) incidence rate.

	HFMD incidence rate (per 100,000)			
	Low (7–149)	Middle (150–249)	High (250–420)	Overall
Number of counties (%)	85 (59.9)	39 (27.5)	18 (12.6)	142
Number of cases (%)	50,955 (36.8)	48,475 (35.0)	39,163 (28.2)	138,593
Number of outbreaks (%)	32 (29.6)	47 (43.5)	29 (26.9)	108
Median number of cases peroutbreak (P_25_, P_75_)	12 (11,15)	14 (12,18)	13 (11,16)	13 (11,17)

Using data from all counties to determine the optimal alerting threshold for C1, C2 and C3, we found that the optimal thresholds for C1, C2 and C3 were 0.4, 0.4, and 0.5, respectively. When using these thresholds to apply the three algorithms to the regions with high disease incidence, C1 and C2 had the highest sensitivity (96.6%), C3 had the highest specificity (88.2%), and the three methods had the same TTD (3 days) (Table 2). For the regions with middle disease incidence, C1 and C2 had the highest sensitivity (91.5%), while C3 had the highest specificity (88.5%), and the three methods had the same TTD (2 days). For the regions with low disease incidence, C1 and C3 had the highest sensitivity (96.9%), C3 had the highest specificity (89.0%), and three methods had the same TTD (0.5 day).

**Table 2:** The number of signals, sensitivity, specificity and time to detection for C1, C2 and C3 algorithms, based on the optimal thresholds identified by employing the data for all counties.

The regions with different level of HFMD incidence rate*	Algorithm	Optimal threshold	No. signals	No. detectedoutbreaks	Sensitivity(%)	Specificity(%)	Time to detection (d)
High level	C1	0.4	600	28	96.6	86.8	3
	C2	0.4	564	28	96.6	87.7	3
	C3	0.5	543	26	89.7	88.2	3
Middle level	C1	0.4	1132	43	91.5	87.4	2
	C2	0.4	1095	43	91.5	88.0	2
	C3	0.5	1049	42	89.4	88.5	2
Low level	C1	0.4	1758	31	96.9	87.9	0.5
	C2	0.4	1665	30	93.8	88.6	0.5
	C3	0.5	1600	31	96.9	89.0	0.5
Overall	C1	0.4	3490	102	94.4	87.6	1
	C2	0.4	3324	101	93.5	88.3	1
	C3	0.5	3192	99	91.7	88.8	1

* The low level of incidence rate ranged from 7 to 149 per 100,000, the middle level of incidence rate being 150–249 per 100,000, and the high level of incidence rate being 250–420 per 100,000.

When using only data from counties within a single incidence class to determine the optimal alerting threshold for each algorithm, for regions with a high disease incidence, C1, C2 and C3 all had an optimal threshold of 0.3. Applying the algorithms to high-incidence counties with this threshold resulted in a TTD of 2 days, with C1 having the highest sensitivity (100%) and C2 having the highest specificity (86.8%) (Table 3). For the regions with a middle disease incidence, C1, C2 and C3 all had an optimal threshold of 0.3, which resulted in a TTD of 1 day, with C1 having the highest sensitivity (100%) and C2 having the highest specificity (87.2%). For the regions with low disease incidence, the optimal thresholds for C1, C2 and C3 were 0.7, 1 and 1.3, respectively. The three methods had the same TTD of 0.5 days, with C1 having the highest sensitivity (96.9%) and C3 having the highest specificity (92.6%).

**Table 3:** The number of signals, sensitivity, specificity and time to detection for C1, C2 and C3 algorithms, based on the optimal thresholds identified separately for the 3 categories of region with different level of HFMD incidence rate.

The regions with different level of HFMD incidence rate*	Algorithm	Optimal threshold	No. signals	No. detected outbreaks	Sensitivity(%)	Specificity(%)	Time to detection (d)
High level	C1	0.3	653	29	100	85.7	2
	C2	0.3	605	28	96.6	86.8	2
	C3	0.3	611	28	96.6	86.6	2
Middle level	C1	0.3	1206	47	100	86.6	1
	C2	0.3	1160	46	97.9	87.2	1
	C3	0.3	1167	46	97.9	87.1	1
Low level	C1	0.7	1482	31	96.9	89.8	0.5
	C2	1	1165	29	90.6	92.1	0.5
	C3	1.3	1084	29	90.6	92.6	0.5
Overall	C1	0.3/0.3/0.7	3341	107	99.1	88.1	1
	C2	0.3/0.3/1.0	2930	103	95.4	89.7	1
	C3	0.3/0.3/1.3	2862	103	95.4	90.0	1

* The low level of incidence rate ranged from 7 to 149 per 100,000, the middle level of incidence rate being 150–249 per 100,000, and the high level of incidence rate being 250–420 per 100,000.

Comparing algorithm performance when the optimal thresholds were determined from all counties, as opposed to within each incidence class, the sensitivity of outbreak detection was higher when using only data from one incidence class (C1: from 94.4% to 99.1%, C2: from 93.5% to 95.4%, C3: from 91.7% to 95.4%), as was specificity (C1: from 87.6% to 88.1%, C2: from 88.3% to 89.7%, C3: from 88.8% to 90.0%), The number of signals was statistically significantly decreased when using only data from one incidence class (the percentage of signals reduction for C1∶4.3% (p<0.001), for C2∶11.9% (p<0.001), for C3∶10.3% (p<0.001)), while maintaining a consistent TTD (1 day).

## Discussion

The results of this study demonstrate that adopting optimizing surveillance alert thresholds by incidence category can improve aberration detection performance as compared to using the same alert threshold across all regions. In particular, for the EARS algorithms applied to HFMD data from counties in Shandong province we observed the same TTD, but higher sensitivity and specificity when alert thresholds were optimized within three incidence categories.

Our findings may be explained in part by the observation that the number of cases and the number and scale of outbreaks differed greatly among regions from different incidence categories. These factors help to explain why the optimal threshold of an algorithm may differ across regions with unequal disease incidence. In other words, the optimal alert threshold for an aberration detection method across all region is a compromise of sorts. A gain in detection accuracy can be realized by further optimizing the alert threshold for groups of sub-regions with similar disease incidence.

An important strength of this study is the use of a large amount of real surveillance data with validated case and outbreak reports. This study is the first to suggest a straightforward method for improving the accuracy of outbreak detection algorithm in a large area by optimizing alerting thresholds within incidence categories.

One limitation of our study is that we used only one disease as an example and it is possible that our results will not generalize to other diseases with a low incidence. In our study we divided counties in Shandong province into three incidence categories to reflect natural groupings in the data, but without considering other factors, such as the differences of population, case report timeliness and completeness, and the characteristics of seasonality and weekend effect of surveillance data [20]–[22], which could affect algorithm performance. The objective of this study, however, was to explore the influence of the variation in incidence rates on algorithm performance, conditional on the observed variation in other factors. Any attempt to simultaneously estimate the absolute effects (and possible interactions) of multiple determinants of outbreak detection would require more extensive adjustment for other factors, but that was not the objective of the study. Also, it is possible that using a greater number of incidence categories and taking into account more characteristics of disease occurring could further improve detection performance and we consider this to be a promising area for future research.

In conclusion, our study illustrates a general method for improving the accuracy of aberration detection algorithms that is potentially applicable broadly to other diseases and regions. Although not measured directly in this study, improvements in the accuracy and timeliness of outbreak detection can have an important impact of the effectiveness of measures to control epidemics and minimize the impact of diseases.
